# Editorial Comment to A case of pyelocalyceal diverticulum urothelial carcinoma that was difficult to distinguish from cystic renal cell carcinoma preoperatively

**DOI:** 10.1002/iju5.12377

**Published:** 2021-09-20

**Authors:** Yasuhide Kitagawa

**Affiliations:** ^1^ Department of Urology Komatsu Municipal Hospital Komatsu Ishikawa Japan

In this issue of *the IJU Case Reports*, Matsuda *et al*. reported a case of pyelocalyceal diverticulum urothelial carcinoma that was difficult to distinguish from cystic renal cell carcinoma.^1^ As the authors described in the paper, the pyelocalyceal diverticulum is rare, and urothelial carcinoma developing in the pyelocalyceal diverticulum is extremely rare.^1^ Additional residual ureterectomy was performed in previous reports summarized in this paper, including this case, which suggested preoperative imaging findings of this disease similar to renal cell carcinoma such as this case.

In this case, the definitive pathological diagnosis was invasive urothelial carcinoma, high‐grade (G3), and pT3. In our previous report, deep invasion in urothelial carcinoma within a pyelocalyceal diverticulum was discussed.^2^ Although there is no consensus on the cause of pyelocalyceal diverticula, one possible cause is derived from dysfunction within sphincters surrounding the calyces that facilitate synchronized filling and emptying.^3^ Such calyceal achalasia results in chronic inefficient emptying, progressive dilatation proximal to the sphincter, and subsequent formation of a diverticulum.^3^ In this situation, possible thinness or loss of the sphincter surrounding calyceal diverticula mucosa may result in tumor invasion across the sphincter muscle layer. Since a high potential of invasion of the renal parenchyma is suspected in this disease, urologists should pay attention to the existence of this disease in clinical practice.

## Conflict of interest

The author declares no conflict of interest.
